# Determination of conifer age biomarker DAL1 interactome using Y2H-seq

**DOI:** 10.48130/FR-2021-0012

**Published:** 2021-07-08

**Authors:** Xi Chen, Qianya Zhu, Yumeng Nie, Fangxu Han, Yue Li, Harry X. Wu, Shihui Niu

**Affiliations:** 1 Beijing Advanced Innovation Center for Tree Breeding by Molecular Design, National Engineering Laboratory for Tree Breeding, College of Biological Sciences and Technology, Beijing Forestry University, Beijing 100083, PR China; 2 Umeå Plant Science Centre, Department of Forest Genetics and Plant Physiology, Swedish University of Agricultural Sciences, Linnaeus väg 6, SE-901 83, Umeå, Sweden

**Keywords:** Ageing, Conifer, *DAL1*, Proteins-interaction, Y2H-seq

## Abstract

Age is a sophisticated physiological signal that ensures the sequence of different developmental stages in organisms. The regulation of ageing pathways appears to differ between gymnosperms and angiosperms. We previously identified *DAL1* as a conserved conifer age biomarker that plays a crucial role in the transition from vegetative to reproductive life-history phases in pines. Therefore, elucidating the specific interaction events related to DAL1 is key to understanding how age drives conifer development. Large-scale yeast two-hybrid (Y2H) analysis followed by next-generation high-throughput sequencing (Y2H-seq) allowed us to identify 135 PtDAL1 interacting proteins in *Pinus tabuliformis*. Our study found that PtDAL1 interacting proteins showed an ageing-related module, with sophisticated interacting networks composed of transcription factors (TFs), transcriptional regulators (TRs), and kinases. These interacting proteins are produced in response to a variety of phytohormones and environmental signals, and are likely involved in wood formation, needle development, oleoresin terpenoids biosynthesis, and reproductive development. In this study, we propose a novel regulation model of conifer ageing pathways whereby PtDAL1 coordinates different environmental stimuli and interacts with corresponding proteins to regulate appropriate development.

## INTRODUCTION

Ageing covers every developmental stage of plants and includes vegetative growth, maturity (beginning reproduction and continuingvegetative growth), senescence, and mortality^[1]^. Some species have short lifespans, while others may be extremely long-lived. Conifers are renowned for their longevity^[2]^ and may even use a special strategy to respond to different environmental stimuli at different ages to ensure their survival and successful reproduction.

The *miR156*-*miR172* module has long been recognized as the key player in ageing pathways in angiosperms^[3−5]^. The miR156 acts as an age biomarker in angiosperms and targets *SPLs* to repress *AP1*, *LEAFY*, *SOC1*, and *miR172* in the shoot apical meristem^[3]^. The expression of miR156 is gradually downregulated with age and releases the expression of the target genes of miR172^[4]^. miR172 represses the expression of *AP2*-like genes to activate *FT*, *AP1*, and *SOC1* further^[5]^. In addition, many transcription factors (TFs) also play an irreplaceable role in ageing regulation (e.g., the *WRKY*, *NAC*, and *MYB* gene families)^[6−9]^. DELLAs repress the expression of *WRKY54*, thereby significantly reducing the expression of its target gene senescence-associated genes to increase plant lifespan^[8]^. Likewise, the *atnap* knockdown mutant delays leaf senescence, while the overexpression of *AtNAP* causes precocious senescence^[7]^. The MYB-bHLH-TTG1 complex regulates the trichome development, which is a phenotype biomarker of age^[9]^. Although these miRNAs and their targets are also found in gymnosperms, they do not show the same age expression trend as they do angiosperms^[10]^. Therefore, whether the miR156-miR172 regulatory module plays a core role in the conifer ageing pathway remains to be confirmed.

In gymnosperms, *AGL6*-like genes have been shown to be involved in ageing pathways and reproductive development. For example, *PaDAL1*, *LaAGL2-1*, and *CjMADS14* display an ascending expressive trend with age in *Picea abies*^[11]^, *Larix kaempferi*^[12]^ and *Cryptomeria japonica*^[13]^. In *L. kaempferi*, some traditional rejuvenating measures such as cutting and pruning^[14]^ can significantly reduce the expression of *LaAGL2-1*^[15]^. In *C. japonica*, the expression of *CjMADS14* can be induced by gibberellin treatment^[13]^. In *Picea abies*, *PaDAL1* causes early flowering and abaxial trichomes initiation in transgenic *Arabidopsis*^[11]^. These results indicate that *AGL6*-like genes have a conserved regulatory function in gymnosperm ageing pathways. However, the regulation mechanisms of *AGL6*-like genes in these ageing pathways are largely unknown.

In our previous study, *PtDAL1*, an *AGL6*-like homologue in *P. tabuliformis*, also displayed a strict age-related expression pattern. Heterologous overexpression of *PtDAL1* also caused extremely early flowering and abaxial trichomes initiating phenotype in transgenic Arabidopsis^[16]^. Moreover, we revealed that PtDAL1 interacted with PtMADS11 to transfer ageing signals to reproduction signals in mature tissues^[16]^. Thus, the formation of heterologous protein complexes with other proteins may be an important mechanism for DAL1 participation in regulating diversified age effects.

Plant ageing pathways are affected by phytohormones, signaling molecules, and TFs, and also respond to environmental cues^[17]^. As age increases, the biochemical substances in plants also experience tremendous changes. In *Platycladus orientalis*, soluble protein content reaches its highest level in 20-year-old trees^[18]^ and protein variety declines with age^[19]^. Additionally, the carbonylation^[17]^, glycosylation^[17]^, and tyrosine phosphorylation^[20]^ of proteins in plant cells increases with age. The accumulation of calcium, manganese, and even lignin and tannins in needles, also shows an age-dependent relationship^[21]^. The dynamic expression of TF coding genes has a more active contribution in transitions of biological processes at the senescence stage^[22,23]^. Although these previous studies are valuable for understanding key regulatory pathways, analyses were mostly limited to illustrating the effects of biological processes during the senescence phase at the chemical and protein levels.

Most biological processes are regulated by recruiting different proteins to form a protein complex. MADS-box family TFs also function by forming tetramers^[23]^. Screening interacting proteins is a technique that can be applied to understand the regulatory mechanism of *PtDAL1* in ageing pathways. Immunoprecipitation, pull-down assays, and yeast two-hybrid (Y2H) assays are commonly used for the screening of interacting proteins^[24]^. Among them, the Y2H system is the most accessible method for large-scale screening^[25,26]^. Unfortunately, the traditional Y2H system has limitations including low plasmid extraction and high sequencing costs^[25,27]^.

Here, we developed a Y2H-seq method that combined Y2H with next-generation sequencing techniques. With this optimized and efficient new technology, we screened the PtDAL1 interactome at the entire transcriptome scale. We show that PtDAL1 recruits relevant proteins to regulate growth or reproduction at different age phases. We propose a model whereby *PtDAL1* responds to different environmental stimuli and interacts with the relevant proteins to regulate plant development through the ageing pathway. Our data provide the foundation for understanding ageing regulatory mechanisms in conifers.

## RESULTS

### The efficiency of Y2H-seq compared to traditional Y2H

**Figure 1:**
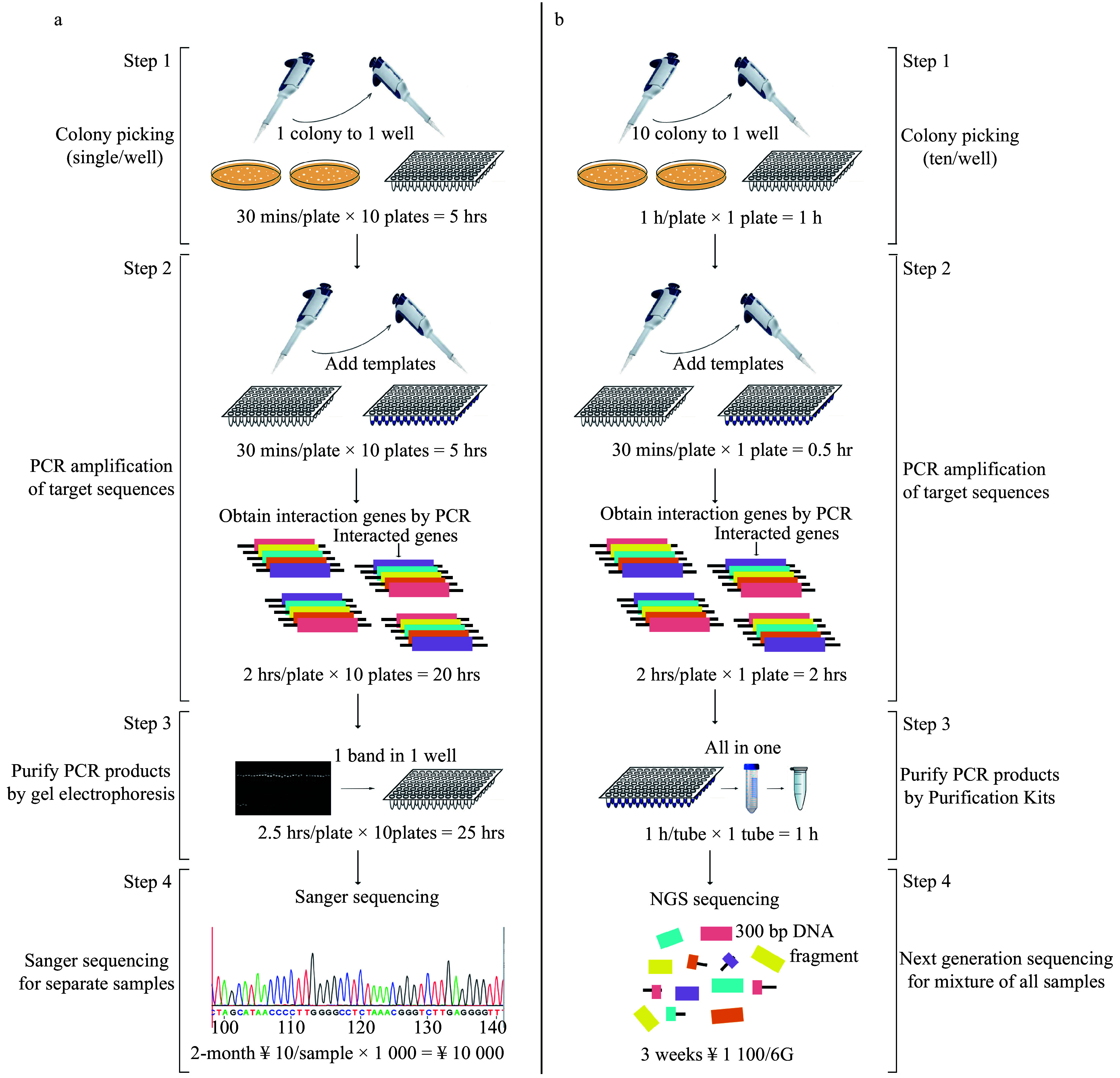
Flowchart of traditional Y2H and Y2H-seq. (a) Traditional Y2H, (b) Y2H-seq technology.

To obtain global PtDAL1 interacting proteins, the Y2H system was applied to screen a cDNA library from different age samples of *P. tabuliformis*. In a traditional Y2H assay (Fig. 1a), single clones would need to be individually selected to extract plasmids for PCR; this process would need to be repeated several times. Then, we would need to individually purify the PCR products and sequence the purified products using Sanger sequencing. These procedures are highly inefficient to complete a large-scale project and are very uneconomical due to the high redundancy of the results. We optimized this technology by combining the Y2H and next-generation sequencing (NGS) as an Y2H-seq assay (Fig. 1b) that requires carrying out PCR one time only. We picked 10 single clones and mixed them in 50 μl ddH_2_O, omitting the plasmid extraction. Next, all the PCR products were mixed in one tube for purification. This change decreased our experimental time and cost significantly. Finally, we sequenced the purified products using NGS sequencing^[28]^; this was chosen instead of Sanger sequencing, as Sanger cannot use a mixed sample. The optimized system reduced the whole experimental time by 66% and reduced cost by 89%. The optimized technology allowed us to identify the interacting proteins thoroughly.

### Global interactome of age biomarker PtDAL1 in *Pinus tabuliformis*

Based on Y2H-seq, we screened the PtDAL1 interactome at the whole-transcriptome scale. We identified 135 PtDAL1 non-redundant interacting proteins from 1000 events (Table 1), including 21 TFs, two TRs, and two kinases, as well as enzymes, heat shock proteins, and RNA binding proteins.

**Table 1:** PtDAL1 interacting proteins annotation.

Gene_ID\Symbol	At_homo	e-value	At_gene_symbol	TF/TR/PK	EST count
Pt0G09550	AT1G74310	1.32E-39	AtHSP101		33,639
Pt0G15520	AT4G38510	0	AtVAB2		34,522
Pt0G21450	AT5G08640	2.8E-125	AtFLS1		28,899
Pt0G22130	AT4G37480	6.7E-137			22
Pt1G04610\PtDAL9	AT2G45660	3.41E-65	AGAMOUS-LIKE 20 (AtSOC1)	TF	66,859
Pt1G04670\PtDAL19	AT2G45660	8.53E-57	AGAMOUS-LIKE 20 (AtSOC1)	TF	573
Pt1G04680		−			31
Pt1G04770\PtMADS11	AT2G45660	2.34E-53	AGAMOUS-LIKE 20 (AtSOC1)	TF	688
Pt1G04910\PtMADS13	AT2G45660	7.91E-61	AGAMOUS-LIKE 20 (AtSOC1)	TF	257
Pt1G04940\PtMADS26	AT2G45660	1.37E-61	AGAMOUS-LIKE 20 (AtSOC1)	TF	78
Pt1G21750	AT5G42270	0	VARIEGATED 1 (VAR1)		116,184
Pt1G21760	AT5G42270	0	VARIEGATED 1 (VAR1)		40
Pt1G26900	AT1G01090	0	PDH-E1 ALPHA		330
Pt1G31120	AT2G05790	5.67E-74			57
Pt1G38270	AT2G05790	1.95E-81			13,837
Pt1G38280	AT2G05790	1.95E-81			7,453
Pt1G42680	AT5G63480	1.18E-37	MEDIATOR 30 (MED30)	TR	210,880
Pt1G50870	AT5G55160	6.87E-45	AtSUMO2		26
Pt1G53330	AT4G31200	1E-178			156,474
Pt1G54050	AT2G21660	8.22E-40	GLYCINE RICH PROTEIN 7 (AtGRP7)		11
Pt1G56320		−			52
Pt1G57520	AT3G05530	0	RPT5A		201,789
Pt1G58710	AT5G55160	5.72E-33	AtSUMO2		48,291
Pt1G70570		−			13
Pt2G09370	AT1G09070	4.32E-36	AtSRC2		18,394
Pt2G09380		−			11,074
Pt2G09430		−			62,709
Pt2G12420	AT2G46210	0	AtSLD2		388
Pt2G22810\PtLEAFY	AT5G61850	2.7E-115	LEAFY 3 (LFY3)	TF	10
Pt2G28740\PtbHLH143	AT1G59640	1.27E-38	BIG PETAL (BPE); ZCW32	TF	97
Pt2G29110	AT5G14040	0	MPT3		52,616
Pt2G29160	AT5G55180	6.73E-42			30
Pt2G29170		−			36
Pt2G29410		−			133
Pt2G36780	AT3G09970	3.2E-138	RHIZOBIALE-LIKE PHOSPHATASE 2 (RLPH2)		72
Pt2G41770\PtDAL10	AT1G26310	4.84E-47	CAULIFLOWER (CAL)	TF	4
Pt2G48290		−			10
Pt2G58590	AT1G63000	0	UER1		56,441
Pt2G66560		−			16,151
Pt2G71640	AT1G75290	2.28E-97			42,597
Pt2G72330		−			669
Pt2G72340		−			33
Pt2G73010		−			16,500
Pt3G00960		−			16
Pt3G26100	AT2G29630	0	THIAMINC (THIC)		136,084
Pt3G52020	AT1G47710	2.7E-133	AtSERPIN1		853
Pt3G52520		−			30
Pt3G54180\PtMADS49				TF	1
Pt3G61960	AT4G32460	9.17E-63	AtHA1-1		119
Pt4G01770\PtTPS_di41	AT4G16730	1.72E-72	TERPENE SYNTHASE 02 (TPS02)		25
Pt4G02100		−			91
Pt4G06200		−			551,090
Pt4G06880	AT4G32470	4.21E-52			2,220,400
Pt4G23680		−			575
Pt4G35470	AT5G21060	2.6E-50			554
Pt4G38090		−			31
Pt4G39400	AT5G09760	1.6E-155			117
Pt4G40470	AT3G05530	1.46E-88	RPT5A		21,709
Pt4G40480	AT3G05530	3.5E-127	RPT5A		21,180
Pt4G42010	AT1G74310	9.99E-92	AtHSP101		1,847
Pt4G47930		−			49
Pt4G64210	AT2G04160	4.49E-46	AUXIN-INDUCED IN ROOT CULTURES 3 (AIR3)		10
Pt5G00580	AT1G21980	4E-146	AtPIP5K1; AtPIPK1	PK	98,948
Pt5G06310	AT3G46740	0	TOC75-III; MAR1		349
Pt5G19560	AT1G75030	2.04E-95	AtLP-3		17,623
Pt5G20480\PtGATA5				TF	1
Pt5G40090		−			12
Pt5G50950	AT3G05530	5.4E-143	RPT5A		85,346
Pt5G54180	AT5G43060	0	ESPONSIVE TO DEHYDRATION 21B (RD21B)		27
Pt5G57130	AT5G43060	5.3E-131	ESPONSIVE TO DEHYDRATION 21B (RD21B)		41
Pt5G62470\PtDAL4	AT2G45660	1.96E-67	AGAMOUS-LIKE 20 (AtSOC1)	TF	1,003,270
Pt5G62640\PtDAL35	AT2G45660	2.36E-67	AGAMOUS-LIKE 20 (AtSOC1)	TF	182,559
Pt6G17610	AT2G21660	7.65E-43	AtGRP7		2,751
Pt6G34620	AT2G21660	7.48E-41	AtGRP7		518
Pt6G35050\PtDAL1	AT2G45650	1.44E-81	AGAMOUS-LIKE 6 (AGL6)	TF	126
Pt6G37350\PtMADS5	AT2G22540	3.69E-59	SHORT VEGETATIVE PHASE (SVP)	TF	9
Pt6G39740	AT3G07320	8.7E-174			290,696
Pt6G41700	AT4G25200	3.8E-36	ATHSP23.6-MITO		157
Pt6G44930		−			23
Pt6G51040	AT4G26830	7E-145			202,392
Pt6G51050	AT2G05790	0			4,185,410
Pt7G03490	AT3G04880	2.3E-137	DNA-DAMAGE-REPAIR/TOLERATION 2 (DRT102)		224
Pt7G14660	AT5G42800	9.3E-164	DFR		2,048
Pt7G15050	AT5G06570	1.48E-37			21,889
Pt7G15060	AT1G68620	3.89E-50			73,081
Pt7G20560	AT3G23900	4.2E-120	IRR		4,284
Pt7G21710		−			6,227
Pt7G24630		−			14
Pt7G32610	AT3G05500	3.24E-70	SRP3; LDAP3		12,253
Pt7G48580	AT5G55180	1.85E-85			53
Pt7G50230	AT3G45310	1.3E-174			32
Pt7G50300	AT3G45310	2E-174			4,816
Pt7G58880	AT5G43060	9.3E-122	RD21B		8,270
Pt8G00820	AT1G18250	4.7E-107	ATLP-1		7,596
Pt8G00840	AT1G18250	4.5E-107	ATLP-1		94,972
Pt8G04720	AT5G55180	8.1E-82			135
Pt8G04770	AT5G55180	1.27E-88			5,885
Pt8G15270\PtDPL1	AT1G14920	6.5E-170	RESTORATION ON GROWTH ON AMMONIA 2 (RGA2)	TF	9
Pt8G15340\PtDPL2	AT2G01570	1.1E-128	REPRESSOR OF GA1-3 1 (RGA1)	TF	64
Pt8G16740		−			27
Pt8G19120	AT5G46290	0	3-KASI		101
Pt8G35940	AT5G47000	4.2E-125			75,498
Pt8G46580	AT5G43060	0	RD21B		14,683
Pt8G46590	AT5G43060	0	RD21B		186,050
Pt8G48720	AT3G05530	1.5E-143	RPT5A		13,449
Pt9G05110		−			415
Pt9G05120		−			28
Pt9G10750		−			11,338
Pt9G10800		−			8,111
Pt9G21000	AT5G38660	1.37E-61	APE1		87,565
Pt9G28260		−			372
Pt9G36230	AT4G23160	1.7E-116	CYSTEINE-RICH RLK 8 (CRK8)	PK	17
Pt9G39600	AT4G12070	1.1E-151			95,337
Pt9G40380	AT1G55000	4.51E-87			26,718
Pt9G46960	AT2G17420	0	NTR2; AtNTRA		61,919
PtJG05540\PtNEEDLY	AT5G61850	1.22E-88	LEAFY 3 (LFY3)	TF	24
PtJG13360	AT1G54320	7.1E-173			12
PtJG38830	AT1G24510	0	CCT5		30,744
PtJG40190	AT3G04880	1.16E-53	DRT102		271
PtQG03450/PtLWD1	AT1G12910	0	AtAN11		41,358
PtQG06810\PtSMU	AT2G26460	2.8E-113	SUPPRESSORS OF MEC-8 AND UNC-52 2 (SMU2)		1
PtQG22390	AT4G27670	1.21E-64	HEAT SHOCK PROTEIN 21 (HSP21)		22,925
PtXG01260	AT1G18640	2.4E-115	PSP1		66,835
PtXG01380	AT1G18640	1.6E-115	PHOSPHOSERINE PHOSPHATASE 1 (PSP1)		49
PtXG01810	AT5G21060	1.92E-53			30
PtXG11800		−			43
PtXG23600\PtbZIP59	AT4G38900	1.05E-89	BASIC LEUCINE-ZIPPER 29 (BZIP29)	TF	1
PtXG26610\PtLUG5	AT4G32551	0	LEUNIG (LUG)	TR	7
PtXG33640\PtRGA	AT2G01570	2.2E-172	REPRESSOR OF GA1-3 1 (RGA1)	TF	5
PtXG33750\PtDPL3	AT2G01570	2.2E-172	REPRESSOR OF GA1-3 1 (RGA1))	TF	133
PtXG35990		−			56
PtXG36010	AT4G26830	6.01E-90			37
PtXG37060	AT3G44620	3.69E-86			106,593
PtXG44840\PtDAL21	AT1G24260	4.83E-48	AGAMOUS-LIKE 9 (AGL9); SEPALLATA3 (SEP3)	TF	13
PtXG49760	AT4G29040	0	RPT2a		92,990

**Figure 2:**
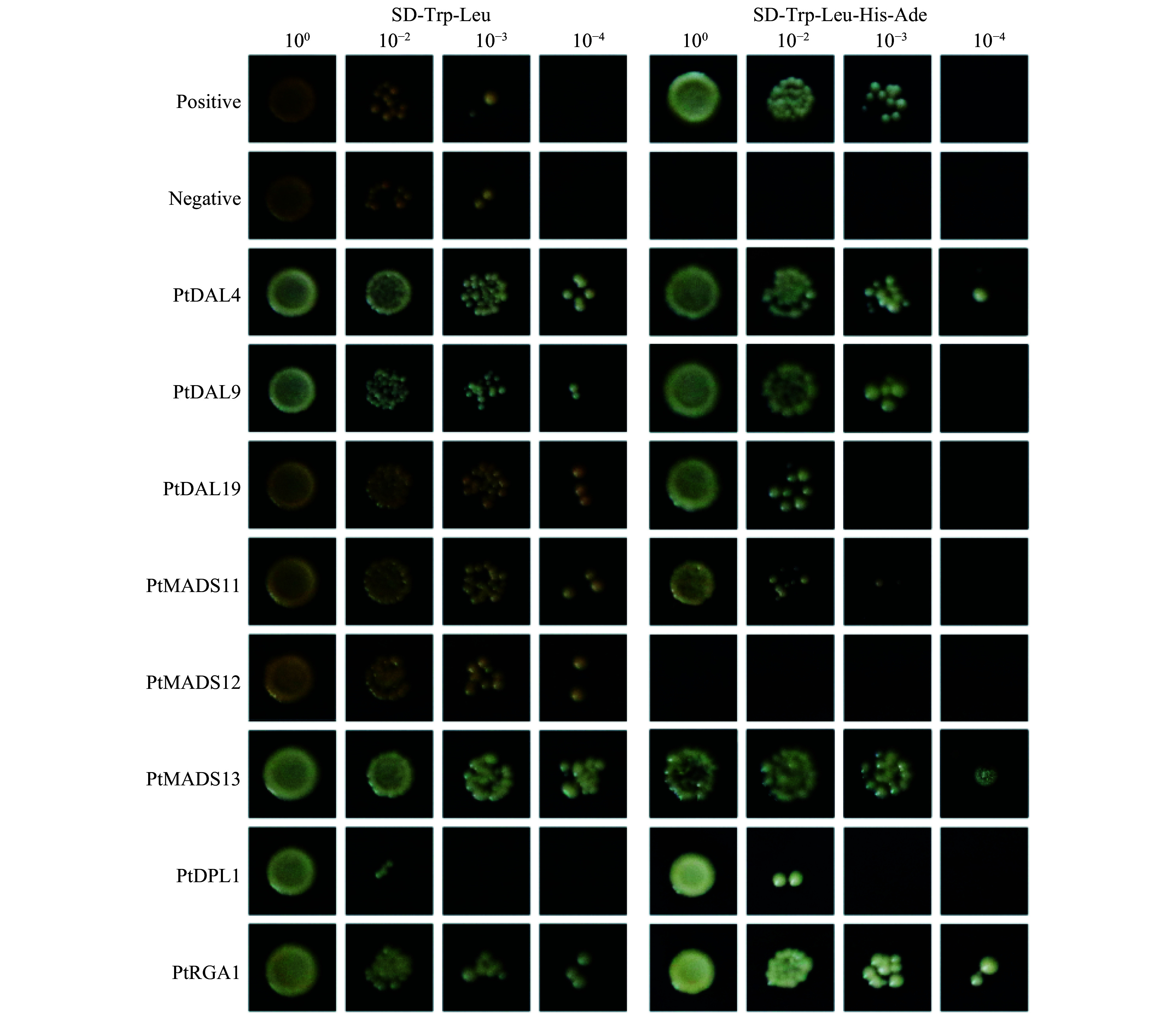
Y2H verification of PtDAL1 interacting proteins. Yeast liquid dilution concentration: 10^0^, 10^−2^, 10^−3^, 10^−4^ (from left to right).

A total of 12 MADS-box proteins and four DELLA proteins were screened out as PtDAL1 interacting proteins (DIPs). Due to their important role in reproductive development and hormone signal transduction, we verified the interaction between them and DAL1 point-to-point through Y2H assay (Fig. 2). In Y2H, the majority of the point-to-point results were consistent with the screening results. Only the PtMADS12 produced a negative result, which suggests that Y2H-seq has comparable accuracy to the traditional Y2H method (Fig. 2). These results suggest that Y2H-seq is a valid technique, but it requires further verification and research.

### Conifers and Arabidopsis AGL6 homologs have different interaction networks

**Figure 3:**
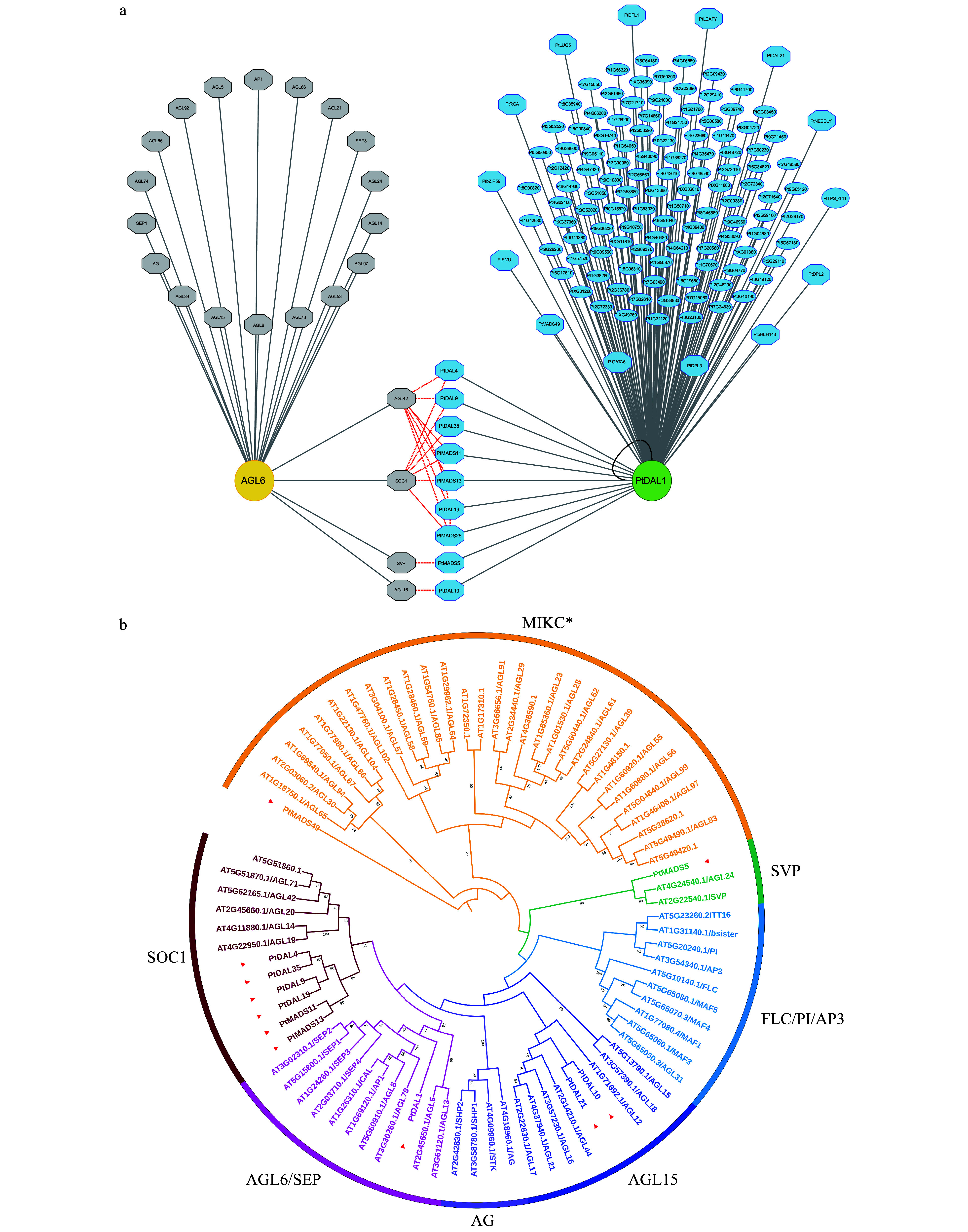
The interactome of PtDAL1 and its Arabidopsis homologue AtAGL6. (a) Gray, AtAGL6 interacted proteins in Arabidopsis. Blue, PtDAL1 interacted proteins in *P. tabuliformis*. Red lines indicate the homologs between Arabidopsis and *P. tabuliformis*. Black lines indicate homologous dimer. Octagon, transcription factors. (b) The phylogenetic analysis of DIPs in MADS-box gene family. Red inverted triangle, DIPs of MADS-box family in *P. tabuliformis*.

*PtDAL1* is a homolog of *AtAGL6* in Arabidopsis. To understand the different functions of conifers and Arabidopsis *AGL6* homologs, we obtained the AtAGL6 interactome from protein interaction databases to compare with the PtDAL1 interactome (Fig. 3a). Interestingly, we found that all of the AtAGL6 interacting proteins belong to the MADS-box family (Fig. 3a). Although the MADS-box proteins are the major components of DIPs, our data showed a more diverse DIP network, including DELLAs (PtRGA, PtDPL1, PtDPL2, and PtDPL3), a bHLH family member (PtbHLH143) and bZIP family member (PtbZIP59), glycosyl hydrolase (Pt6G51050 and Pt2G29160), and floral meristem identity factor (PtNEEDLY) (Fig. 3a). AtAGL6 and PtDAL1 interactomes share some common subfamily members, such as AGL42, SOC1, SVP, and AGL16 (Fig. 3b). This result indicates that the AGL6 homolog interactomes were partially conserved during the seed plant evolution, and these MADS-box proteins may be the core module in the AGL6-mediated regulatory network.

### The expression profiles of PtDAL1 interacting protein coding genes

**Figure 4:**
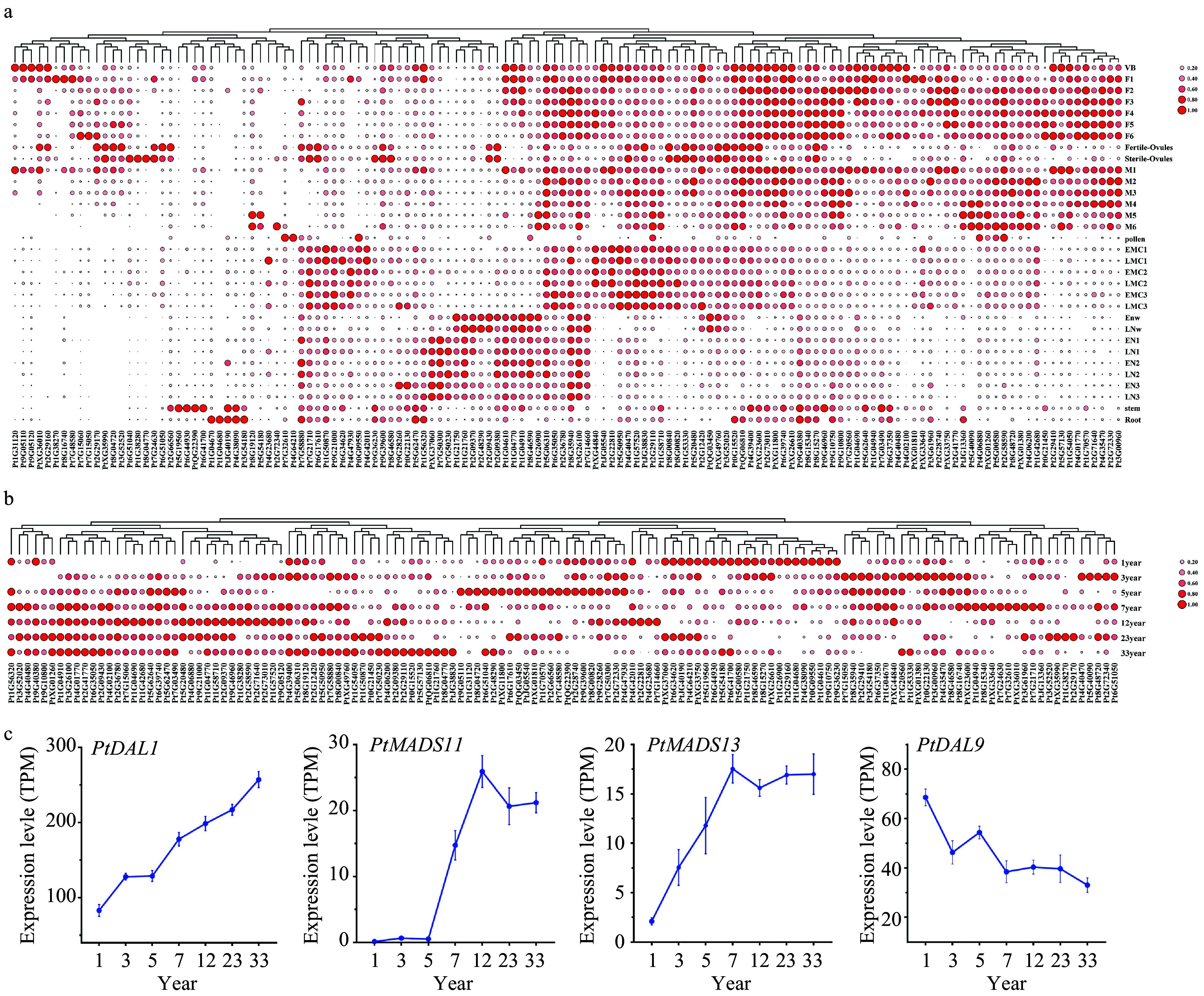
The expression profiles of PtDAL1 interacting proteins. (a) Expression patterns of PtDAL1 interacting proteins in different tissues, (b) expression patterns of PtDAL1 interacting proteins at different ages, (c) expression patterns of PtDAL1, PtDAL9, PtMADS11 and PtMADS13 at different ages.

To reveal the potential biological functions of DIPs, we analyzed the spatial and temporal expression patterns of all DIP coding genes. The results show that most of the DIP genes were expressed in the tissues most similar to *PtDAL1* in *P. tabuliformis* (Fig. 4a, Supplemental Fig. 1). This suggests that *PtDAL1* serves as a global regulatory factor that participates in developing most tissues through interactions with different proteins at different ages. The expression of DIP genes has a significant ageing-related module (Fig. 4b, Supplemental Fig. 2). For example, there are 22 DIP genes that accumulate highly in 1-year-old trees. Similarly, there are 22 DIP genes that accumulate highly in 3-year-old trees, 21 DIP genes that accumulate highly in 5-year-old trees, 18 DIP genes that accumulate highly in 7-year-old trees, and 47 DIP genes that accumulate highly in 12, 23, and 33-year-old trees. These findings suggest that PtDAL1 establishes an age-related genetic program that recruits different proteins corresponding with the developmental stage. *PtMADS11*, *PtMADS13*, and *PtDAL9* all have significant ageing effects similar to *PtDAL1* (Fig. 4c), implying that MADS-box proteins probably play an important role in balancing ageing effects at different stages.

### Promoter *cis* element analysis of PtDAL1 interacting protein coding genes.

**Figure 5:**
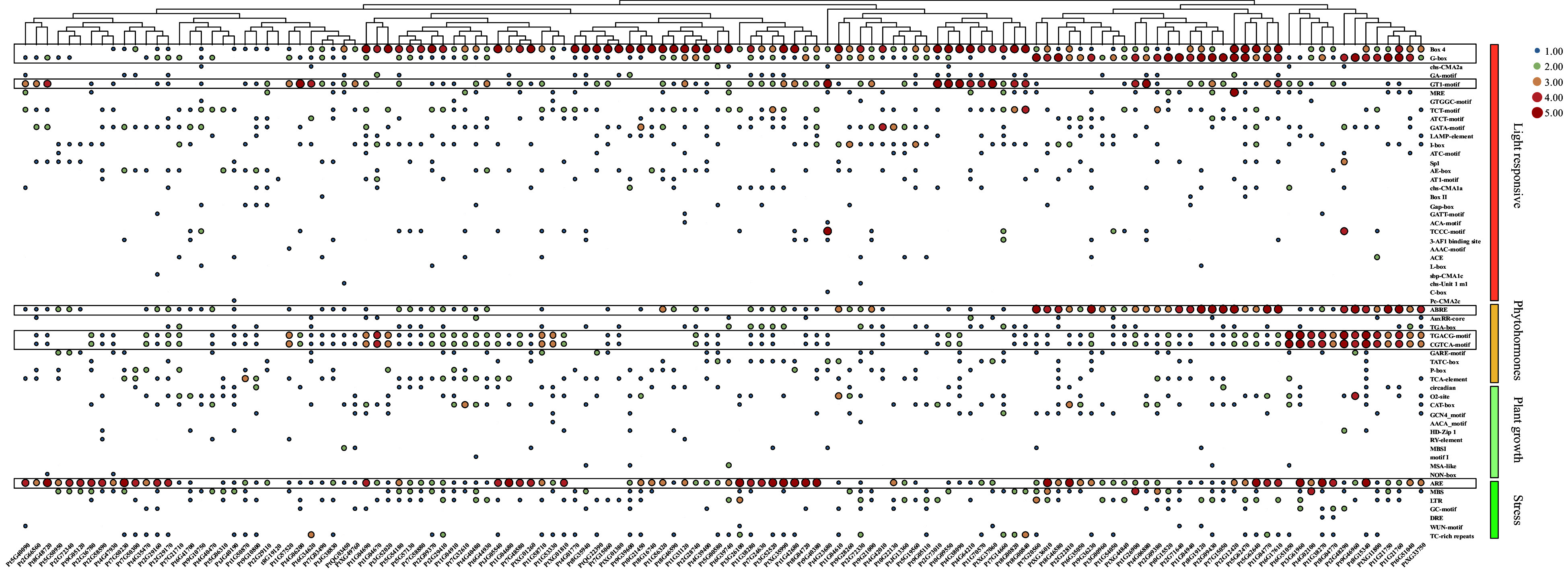
Cis-acting element analysis of PtDAL1 interacting proteins. ABRE, ABA responsive motif; TGACG motif and CGTCA motif, MeJA responsive motif.

To explore the potential regulatory mechanism of DIP genes, the *cis*-elements were identified from their promoters. We found 57 types of *cis*-elements, including 30 light response, 9 phytohormone response, 11 plant growth-related response, and 7 stress response elements (Fig. 5). Among these *cis*-elements, light-responsive elements accounted for the largest proportion, and Box-4, G-box, and GT1-motif were critical elements of light response. For phytohormone response, the ABRE motif related to abscisic acid (ABA) as well as the TGACG motif and CGTCA motif related to the methyl jasmonate (MeJA) were mainly enriched. For stress response, only the ARE motif related to anaerobic induction was enriched. These results indicate the potential role of DIP genes in the circadian rhythm, photosynthesis, ABA-mediated stomatal regulation, MeJA-mediated anthocyanin biosynthesis, and stress response.

### The functions of PtDAL1 interacting proteins

**Figure 6:**
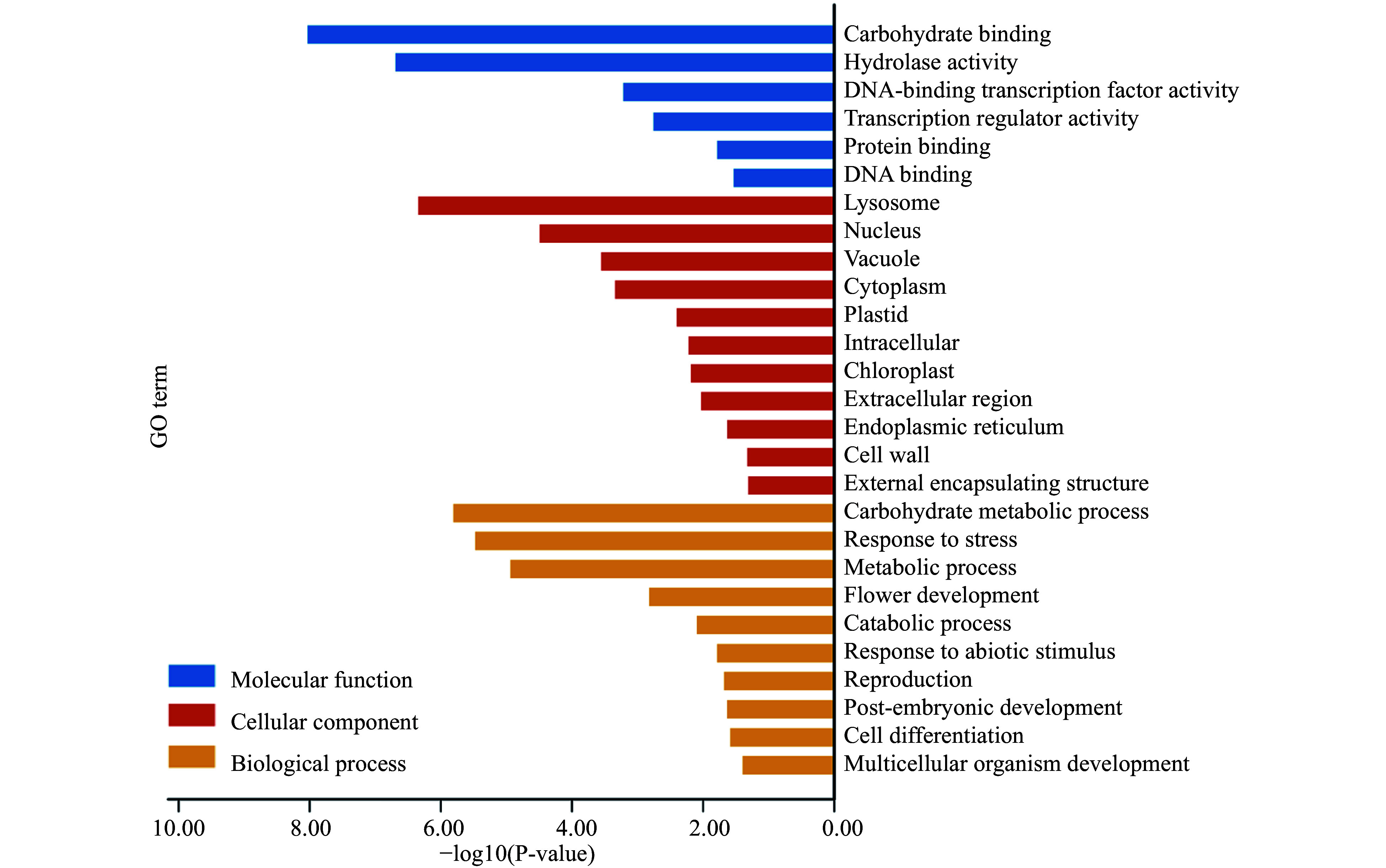
GO functions of PtDAL1 interacted proteins.

The GO enrichment analysis categorized 135 PtDAL1 interacting proteins into three GO subgroups. These interacting proteins were involved in stress response (BP), flower development (BP), abiotic stimulus response (BP), post-embryonic development, lysosome development (CC), and DNA-binding transcription factor activity (MF) (Fig. 6). Considering promoters of DIP genes enriched stress response motif and light-responsive elements, it is not surprising that they participate in stress response and abiotic stimulus response. These results indicate that DIPs play an important role in several growth stages. In addition, the results show that DIPs have a function in flower development, which is consistent with the main functions of the MADS-box gene family^[29]^.

**Figure 7:**
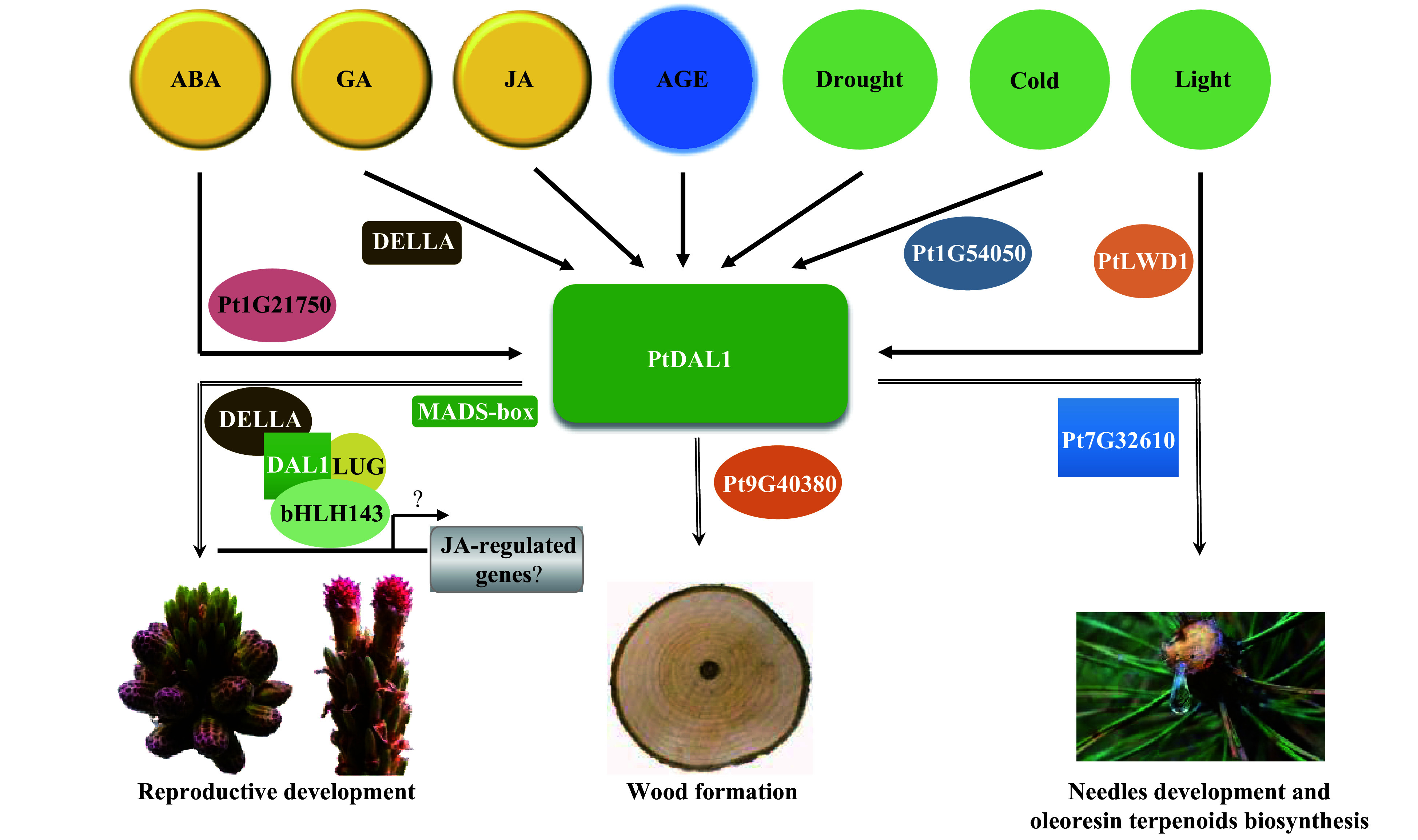
A predictive model for the PtDAL1-mediated growth and development regulation in *P. tabuliformis.*

According to the results described above, we present a working model for PtDAL1 as a core node in ageing networks. Our results suggest that PdDAL1 interacts with proteins selectively under phytohormone, light, and stress conditions to regulate plant vegetative growth, reproductive development, and oleoresin terpenoid biosynthesis (Fig. 7). In summary, PtDAL1 is an ageing signal integrator, combining environmental signals and endogenous signals to mediate the balance of vegetative and reproductive development at different growth stages.

## DISCUSSION

The conifer undergoes a series of developmental processes throughout its whole lifespan, requiring the coordination of phytohormones, TFs, and a variety of environmental cues. In this study, we explored which proteins PtDAL1 interacted with to play a phased regulatory role in the ageing pathway of *P. tabuliformis*. Interrogation of the PtDAL1 interactome in different ages of *P. tabuliformis* provided a comprehensive resource for understanding transcriptional programs underlying the age-dependent utilization of diverse compositions of protein polymers.

Identifying interacting proteins by traditional Y2H technique requires extracting plasmids from yeast to PCR for sequencing^[25,27]^. In our study, we found that the difficulty of sample identification and the cost of sequencing will rise exponentially as hundreds to thousands of interacting events are screened. Many technological improvements have enabled the next generation of high-throughput sequencing technology^[28]^. In the Y2H-seq we present in this study, we omitted several costly and time-consuming steps, reducing experimental time and cost significantly. We selected TFs with important functions among the DIPs, verifying the reliability of the screening results. Most of the tested DIPs interacted with PtDAL1, except PtMADS12 (Fig. 2). This false positive result may be caused by short reads in the building of the sequencing library because the sequences of *PtMADS11*, *PtMADS12*, and *PtMADS13* are too similar to distinguish. In conclusion, Y2H-seq is a convenient and reliable method that can be used to supplement common Y2H.

Comparing the interactome between PtDAL1 and AtAGL6, we found that there are more DIPs than interacting proteins of AtAGL6, indicating that PtDAL1 may be associated with more biological functions than AtAGL6. In addition, the PtDAL1 and AtAGL6 interactome share some common interactors belonging to MADS-box subfamilies, including AGL42, SOC1, SVP, and AGL16 (Fig. 3a). This result suggests that the function of PtDAL1 is probably conservative in reproductive development. *AGL42* and *SOC1* are positive regulators to accelerate flowering times^[30−34]^, while *SVP* and *AGL16* have a negative impact on reproductive development^[35−38]^. PtDAL1 may also interact with AGL42/SOC1-like proteins or AGL16/SVP-like proteins to balance vegetative growth and reproduction. However, there is not enough conclusive evidence showing the function of *AtAGL6* in ageing pathways in Arabidopsis. Moreover, age-related genes and microRNA in angiosperms do not show conspicuous expression differences with age^[10]^, such as *WRKY*^[6]^, *NAC*^[39]^, and *MYB*^[22]^ family genes as well as *miR156*^[3,4]^, and *miR172*^[3]^. These findings indicate that PtDAL1 is probably the core regulator of the unique ageing regulatory system in gymnosperms.

Phytohormones are the prominent factors in ageing pathways. In angiosperms, salicylic acid (SA), jasmonic acid (JA), abscisic acid (ABA), and ethylene are all involved in ageing regulation^[1,22]^. JA response decays with plant age, but defensive substances are accumulated^[40−42]^. In conifers, researchers also found that the expression levels of ethylene increased with age^[18]^. In our study, we found that JA and ABA were also involved in ageing regulation (Fig. 5), indicating that the effects of some phytohormones in ageing are conserved. We also found many light-related proteins in DIPs, as *TOC1* connects the circadian and ageing pathways in angiosperms^[43]^, suggesting light may be a factor in ageing regulation. Thus, further exploration of the mechanisms of ageing pathways and biological processes is warranted at the molecular level.

In Arabidopsis, DELLAs-WD40-bHLH-MYB protein polymer plays a key role in JA-mediated regulation of trichome development^[9]^. *LEUNIG (LUG)* is a repressor having the WD40-repeat domain, which interacts with different proteins to regulate critical developmental processes in angiosperms. Some of these processes include promoting adaxial cell identity in leaves and embryonic shoot apical meristem initiation^[44]^ as well as flower development^[45]^. In addition, *LUG* is also involved in JA response by regulating *MYELOCYTOMATOSIS 2* (*MYC2*) targets^[40]^. *bHLH* family genes also regulate flower size^[46,47]^ and trichome development through JA response^[48]^. In our data, *PtLUG5*, *PtbHLH143*, and DELLAs were highly expressed in the 5-year-old tree. Thus, we suggest PtDAL1 will interact with DELLAs, PtLUG5, and PtbHLH143 to form polymers that regulate reproductive development. This proposition requires further verification.

Various biological processes of the interactome resource of PtDAL1 in *P. tabuliformis* can be analyzed to address a broad spectrum of questions on conifer lifespan. For example, the conifer ageing effect is the most important limitation of somatic embryogenesis and organogenesis^[49]^. Thus, a thorough understanding of biological networks that delineate conifer ageing is essential to achieve plant regeneration. Further studies to characterize the detailed function of each DIP will provide a more comprehensive network that further extends our understanding of age-associated pathways in gymnosperms. The global DIPs revealed in this study will serve as fundamental resources and facilitate future studies on perceiving the age-dependent processes in perennial forest trees.

## MATERIALS AND METHODS

### Plant materials

We collected new shoots from 1, 3, 5, 7, 12, 23, and 33-year-old trees of *P. tabuliformis* Carr. from a primary clonal seed orchard located in Pingquan City, Hebei Province, China to construct the yeast library.

For tissue-specific expression analysis, we collected needles^[50]^, cones^[51]^, pollen, new shoots, roots, stems^[52]^, and ovule^[53]^. For ageing-specific expression analysis, we collected vegetative shoot apexes^[16]^.

### Yeast two-hybrid (Y2H) and Y2H-seq assay

For the Y2H assay, *PtDAL1* CDS was cloned into the pGBKT7 (BD) (Clontech), and *PtDAL4*, *PtDAL9*, *PtDAL19*, *PtMADS11*, *PtMADS12*, and *PtMADS13* were inserted into pGADT7 (AD)(Clontech). We transferred recombinant plasmids into the yeast strain using Y2H (Weidi Biotechnology Co. Ltd (Shanghai, China)). Transformants were placed on SD-Leu-Trp plates and incubated for 2 d at 30 °C. The interactions were tested on SD-Trp-Leu-His-Ade plates and incubated for 3–4 d at 30 °C.

For the Y2H-seq assay, we constructed the yeast library in OEbiotech company (OEbiotech Co., Ltd (Shanghai, China)). Here, RNA was extracted from new shoots, and then reverse transcription was applied to obtain the whole cDNA in new shoots. Next, the whole cDNA was inserted in pDONR222 and pGADT7-DEST vectors. Finally, the converted expression vectors in the Y187 strain to obtain working liquid. In the meantime, we used pGBKT7-PtDAL1 plasmids to verify self-activation and screen PtDAL1 interacting proteins using the Y2H mating protocol (OEbiotech Co. Ltd (Shanghai, China)). Then, we selected 10 events in one PCR tube with 50 μl ddH_2_O and mixed them. Then, we added 1 μl of mixed liquid to every tube as the template for PCR analyses. To extract as much DNA fragments of DAL1 interacting proteins as possible, we extended the extension time to 3 min if the extension efficiency was 1 kb/min. All PCR products were mixed in a 10 ml tube to purify them. Finally, we used the purified products to sequence by NGS sequencing.

### Construction of interaction network

The AtAGL6 interactome were obtained from the database of EMBL-EBI (http://www.ebi.ac.uk) and BAR (http://bar.utoronto.ca/interactions2). We listed the interacted proteins of PtDAL1 and then constructed the interaction network using Cytoscape 3.6.1 (National Institute of General Medical Sciences (NIGMS)).

### *Cis*-elements analysis

The 2000 bp upstream sequencing of CDS of PtDAL1 interacting proteins in *P. tabuliformis* genome was obtained using TBtools^[54]^. Then, the *cis*-elements of DIP promoters were screened using the PlantCare website (http://bioinformatics.psb.ugent.be/webtools/plantcare/html).

### Heatmap analysis and GO enrichment analysis

We obtained data annotations from the GO database (http://geneontology.org), and functional annotation of these sequences was performed by running BLAST against protein sequences from *Arabidopsis thaliana*. GO enrichment analysis and heatmap of *cis*-elements, tissue-specific expression, and ageing-specific expression were carried out using TBtools^[54]^. Due to the large amounts of data, we used the mean for ageing and tissue-specific expressions (Fig. 4). The entire raw data are provided as supplementary material.

## SUPPLEMENTARY DATA

Supplementary data to this article can be found online.
