# Supplementary material for: Determination of conifer age biomarker DAL1 interactome using Y2H-seq
Source: For Res (Fayettev). 2021 Jul 8;1:12. doi: 10.48130/FR-2021-0012 (PMC11524280; doi:10.48130/FR-2021-0012)
Supplement: Supplementary file 1 — Supplementary data to this article can be found online. [file FR-2021-0012-S1.zip › 10.48130_FR-2021-0012-Suppl-FigureS1 (1).pdf]

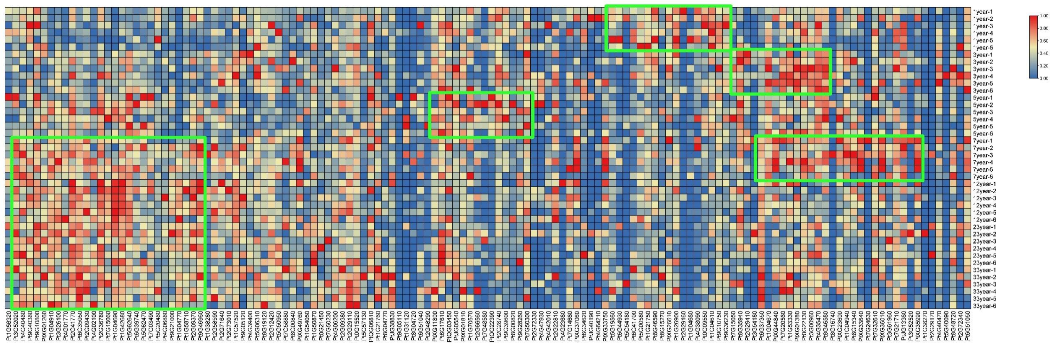

Supplement Figure 2 Expression patterns of PtDAL1 interacting proteins in different ages. Six biological replicates were sequenced for every age.
